# Caveolin-Mediated Internalization of Fmoc-FF Nanogels in Breast Cancer Cell Lines

**DOI:** 10.3390/pharmaceutics15031026

**Published:** 2023-03-22

**Authors:** Giovanni Smaldone, Elisabetta Rosa, Enrico Gallo, Carlo Diaferia, Giancarlo Morelli, Mariano Stornaiuolo, Antonella Accardo

**Affiliations:** 1 IRCCS Synlab SDN, Via Gianturco 113, 80143 Naples, Italy; 2 Department of Pharmacy and Research Centre on Bioactive Peptides (CIRPeB), University of Naples “Federico II”, 80131 Naples, Italy

**Keywords:** nanogel, Fmoc-FF peptide, caveolin, endocytosis, breast cancer cell line, diagnostic agents

## Abstract

Introduction: Hydrogel nanoparticles, also known as nanogels (NGs), have been recently proposed as alternative supramolecular vehicles for the delivery of biologically relevant molecules like anticancer drugs and contrast agents. The inner compartment of peptide based NGs can be opportunely modified according to the chemical features of the cargo, thus improving its loading and release. A full understanding of the intracellular mechanism involved in nanogel uptake by cancer cells and tissues would further contribute to the potential diagnostic and clinical applications of these nanocarriers, allowing the fine tuning of their selectivity, potency, and activity. The structural characterization of nanogels were assessed by Dynamic Light Scattering (DLS) and Nanoparticles Tracking Analysis (NTA) analysis. Cells viability of Fmoc-FF nanogels was evaluated by MTT assay on six breast cancer cell lines at different incubation times (24, 48, and 72 h) and peptide concentrations (in the range 6.25 × 10^−4^ ÷ 5·10^−3^ × wt%). The cell cycle and mechanisms involved in Fmoc-FF nanogels intracellular uptake were evaluated using flow cytometry and confocal analysis, respectively. Fmoc-FF nanogels, endowed with a diameter of ~130 nm and a zeta potential of ~−20.0/−25.0 mV, enter cancer cells via caveolae, mostly those responsible for albumin uptake. The specificity of the machinery used by Fmoc-FF nanogels confers a selectivity toward cancer cell lines overexpressing the protein caveolin1 and efficiently performing caveolae-mediated endocytosis.

## 1. Introduction

In the last decade, nanocarriers like micelles, liposomes, nanofibers, and polymeric nanoparticles have been extensively investigated as potential delivery systems for diagnostic and/or therapeutic agents [1,2,3,4,5,6]. Thanks to their size (diameter of 10–500 nm), nanoparticles display some unique pharmacokinetic features, including rapid clearance via the reticuloendothelial system (RES) and preferential extravasation and accumulation at the site of solid tumors [7]. This latter property results from the enhanced permeability and retention (EPR) effect, which represents a pathophysiological modification involving the vascular endothelial cells of cancer and inflamed areas, where the appearance of fenestrations allows the passage of big molecules [8]. Nanoparticles employ different mechanisms to enter cells [9]. The most recurrent mechanisms are the endocytosis-based pathways that can be further differentiated into five mechanistically distinct classes (clathrin-dependent endocytosis, caveolin-dependent endocytosis, clathrin- and caveolin-independent endocytosis, phagocytosis, and micropinocytosis). As an alternative, nanoparticles can cross the cell plasma membrane via biochemical or physical means to directly enter the cytoplasm by translocation, lipid fusion electroporation, or microinjection [9]. The cell entry pathway is certainly affected by the nanoparticles’ features, including their size and shape, core-corona structure, surface chemistry and charge, hydrophobicity, and mechanical properties [10]. Another important advantage deriving from the employment of nanoparticles is represented by their capability to simultaneously encapsulate multiple copies of different biologically relevant molecules (contrast agents, organic or metallic drugs, and nucleic acids) [11,12,13]. The loading into the inner compartments of the nanoparticles allows them to preserve the bioactive ingredients from potential inactivation they can undergo in the bloodstream. In cancer therapy, the preservation of the drug from degradation together with its controlled release over time are essential to increase the in vivo therapeutic efficacy [14]. Moreover, in some cases, the vehiculation by nanocarriers can represent a solution to overcome the administration difficulties of poorly hydrophilic drugs (e.g., paclitaxel, danazol, and naproxen), which usually require the employment of non-biocompatible organic solvents for their solubilization [15]. On the other hand, the delivery through a nanocarrier allows for significantly improved the in vivo pharmacokinetic and pharmacodynamic properties of the active drug and, consequently, reduced its toxic side effects. In this perspective, in 1995, the Food and Drug Administration (FDA) approved a liposomal doxorubicin (Dox) formulation that was commercially available as Doxil^®^/Caelyx^®^, for the treatment of several pathologies, among them metastatic breast cancer with cardiac risk [16]. Beyond Doxil^®^, an unPEGylated liposomal formulation, named as Myocet^®^, was approved. Indeed, several studies evidenced a significant reduction of myelosuppression and cardiotoxicity of doxorubicin when locally confined into liposomal formulation, together with an increase of the pharmaceutical efficiency, which are related to the different biodistribution of the supramolecular drug [17,18]. After the FDA approval of Doxil^®^, few other nanoparticles reached the market for the cancer treatment [19]. Among them, Eligard^®^ is based on polymeric PLGA (Poly(Lactic-Co-Glycolic Acid)) nanoparticles loaded with leuprolide acetate for the therapy of the prostate cancer. Moreover, there are two formulations for the vehiculation of paclitaxel (Abraxane^®^ and Genexol PM^®^) and one for the vehiculation of Irinotecan (Onivyde^®^) approved for the treatment of the metastatic breast cancer and of pancreatic cancer, respectively.

Recently, nanogels (NGs) have emerged as novel biocompatible systems for the in vivo delivery of drugs and contrast agents [20,21]. Nanogels are supramolecular aggregates with a size in the nanoscale, composed of an interior hydrogel-like network (core) stabilized by an external surfactant coating (shell). They can be obtained for submicronization of macroscopic hydrogels prepared through self-assembly of natural or synthetic polymers (e.g., chitosan, cellulose, agarose, hyaluronic acid, etc.) or, as an alternative, of peptide sequences [22,23,24,25,26]. Due to their biocompatibility, biodegradability, and mild preparation conditions (they do not require the use of extreme pH and temperature values), peptide-based NGs seem to be a promising platform for biomedical applications. Like liposomes and micelles, nanogels are compatible with needle injection, prolonged bloodstream circulation, and accumulation. Moreover, their surface can be easily modified with polymers or with target entities (other peptides, antibodies, or organic molecules) able to recognize the site of interest. NGs differ from liposomes and micelles for their characteristic inner structure, which resembles the entangled fibrillary network of hydrogels. This porous matrix can accommodate a large amount of water and simultaneously interact in a non-covalent way with a plethora of host molecular moieties like aliphatic, alkyl, or aromatic groups of amino acid residues. This feature of peptide-based NGs is probably the most interesting one, since the loading and release properties of the nanovector can be tuned by simply changing the primary sequence of the peptide used for the NG preparation.

In this context, recently we described one of the first stable peptide-based nanogel formulations in the literature [27]. This formulation was prepared using Fmoc-FF (Fmoc-Phe-Phe-OH, *N*^α^-9-fluorenylmethoxycarbonyl-diphenylalanine), which is a well-known low molecular weight hydrogelator able to form self-supporting hydrogels under physiological pH conditions [28]. The resulting Fmoc-FF nanogels demonstrated the capability of encapsulating Dox [29]. Due to the limited number of studies occurring on peptide based NGs, a deep investigation of their cellular behavior is desirable. The study here presented started with the goal of evaluating the cytotoxicity of unloaded nanogels on a panel of breast cancer cell lines. However, in performing the experiments, one of the tested cell lines was more affected than the others by the treatment with unloaded nanogels. To investigate this cargo-independent cell-specific cytotoxicity, we ended up identifying the cellular machinery involved in nanogel intracellular uptake and proving this involves caveolae, mostly those responsible for Human Serum Albumin (HSA) uptake. 

## 2. Experimental Section

Fmoc-FF was purchased from Bachem (Bubendorf, Switzerland). Surfactants used for NG formulations are TWEEN^®^60 (polyethylene glycol sorbitan monostearate) and SPAN^®^60 (sorbitan stearate) and all other chemicals are commercially available from Merck (Milan, Italy), Fluka (Bucks, Switzerland), or LabScan (Stillorgan, Dublin, Ireland). They were all used as received unless otherwise stated. All solutions used in the study were prepared by weight using doubly distilled water as a solvent. Fluorescein-isothiocyanate (FITC-NCS, Merk) powder was used. The effective concentrations were spectroscopically determined in solution by UV-Vis measurements on a Nanodrop 2000c spectrophotometer (Thermo Fisher Scientific Inc., Wilmington, DE, USA) equipped with a 1.0 cm quartz cuvette (Hellma). The molar absorptivity (ε) values used for Fmoc-FF and FITC were 7800 mol^−1^·L·cm^−1^ at 301 nm and 75,000 mol^−1^·L·cm^−1^ at 480 nm, respectively. 

### 2.1. Formulation of Fmoc-FF Nanogels and FITC Loaded Fmoc-FF Nanogels

Fmoc-FF NGs were prepared according to the procedure previously described [29]. Fmoc-FF hydrogel (1.0 wt%) was prepared via the “solvent-switch method” [27]. Fmoc-FF solution (100 mg/mL) was prepared in dimethyl sulfoxide (DMSO). 20 µL of this solution was then diluted with 380 μL of double-distilled water under stirring (5 s). The metastable, opaque suspension was aged at room temperature until transparent and self-supporting gel formation (5 min). The submicronization of Fmoc-FF matrices was achieved via homogenization at 35.000 r/min^−1^ for 5 min into 4 mL of an aqueous solution containing TWEEN^®^60/SPAN^®^60 at a *w*/*w* ratio of 52/48 (3.0·10^−5^ mol) using a MICCRA D-9 homogenizer. The resulting suspension was tip-sonicated for 5 min at 9 W using a tip sonicator (Branson SFX150, Germany). 

FITC-filled NG was prepared as above described. 20 µL of DMSO solution (200 mg/mL) was added to 20 µL of FITC solution in DMSO at a concentration of 12.8 mmol/L. After vortexing, the final DMSO stock was diluted with 360 µL of water. The unloaded FITC was removed from the final formulation via size exclusion chromatography (SEC) using a prepacked gel filtration column (Sephadex G-50) pre-equilibrated with water. The amount of FITC encapsulated was analytically determined via UV-Vis spectroscopy using calibration curves obtained by measuring the absorbance at λ = 492 nm. 

### 2.2. Dynamic Light Scattering (DLS) Measurements

Structural characterization of the nanogel formulations (mean diameter, polydispersity index, and zeta potential, ζ) were achieved by DLS using a Zetasizer Nano ZS (Malvern Instruments, Westborough, MA, USA). Instrumental settings for the measurements and preparation of the samples were previously reported [27]. Size measurements were performed in triplicate, whereas ζ measures were collected as the average of 20 measurements.

### 2.3. Nanoparticles Tracking Analysis (NTA) Measurement

The NTA measurement was done using a Nanosight NS300 (Alfatest, Italy). The Fmoc-FF nanogel formulation (0.1 wt%) was 1000-fold diluted in double-distilled water to a final volume of 1 mL (1 × 10^−4^ wt%). The dilution was done in agreement with the ideal particle per frame value (20–100 particles/frame). The following settings were chosen according to the manufacturer’s software manual (NanoSight NS300 User Manual, MAN0541-01-EN-00, 2017) [30]. 

### 2.4. Cell Culture

The non-tumorigenic epithelial MCF10-a cell line, the human breast cancer cell lines (SKBR3, MDA-MB-231, MDA-MB-361, and MDA-MB-453) and the mouse pre-adipocyte cell line 3T3-L1 were obtained from the SYNLAB SDN Biobank. Cells were grown as previously described [31].

### 2.5. RNA Extraction and RT-PCR Analysis

Total RNA was extracted using the Trizol Reagent following the manufacturer’s instructions (Thermo Fisher Scientific, Waltham, MA, USA) and as previously described [32,33]. 

The following forward (fw) and reverse (rev) primers were used:

- RPS18:
  fw 5′-CGATGGGCGGCGGAAAATA-3′; rev 5-CTGCTTTCCTCAACACCACA-3′
- CyclinA:
  fw 5′-AAATGGGCAGTACAGGAGGA-3′; rev 5′-CCACAGTCAGGGAGTGCTTT-3′
- CyclinB:
  fw 5′-CATGGTGCACTTTCCTCCTT-3′; rev 5′ AGGTAATGTTGTAGAGTTGGTGTCC-3′
- CyclinD:
  fw 5′-GCTGTGCATCTACACCGACA-3′; rev 5′-TTGAGCTTGTTCACCAGGAG-3′
- CyclinE:
  fw 5′-GGCCAAAATCGACAGGAC-3′; rev 5′-GGGTCTGCACAGACTGCAT-3′
- Caveolin 1:
  fw 5’-ACACGGCTGATGCACTGAACTC-3’; rev 5’-GACACACAGGGAAGACCAAGACG-3’

### 2.6. Cell Cycle Analysis

The cycle progression of cells treated with NG formulations was evaluated in MDA-MB-231 cells by flow cytometry (FCM). Cells, incubated with nanogels, were then fixed and stained with propidium iodide (Beckman Coulter, Brea, CA, USA). In the experiment, a minimum of 10.000 single-cell events were acquired. Events and number of cells in G0/G1, S and G2-M phases were acquired using the CXP software (Beckman Coulter) and Kaluza Analysis Software 2.1 (Beckman Coulter) with the Michael Fox algorithm, respectively.

### 2.7. MTT Assays 

Cell viability was assessed by the MTT (3-(4,5-dimethylthiazol-2-yl)-2,5-diphenyltetrazolium bromide) assays (G4000, Promega, Italy). All the tested cell lines were seeded in 96-well plates at a density of 5 × 10^3^ per well. For Fmoc-FF nanogel treatments, cells were incubated for three different times (24, 48, and 72 h) with the nanogel solution at the selected concentrations. Absorbance values were estimated at 490 nm using an automatic plate reader (Victor Nivo, Perkin Elmer, Waltham, MA, USA). Each viability assay was repeated in triplicate. An Analogous protocol was used to evaluate the cell viability of pure TWEEN^®^60/SPAN^®^60 aggregates.

### 2.8. Immunofluorescence Analysis of Fmoc-FF Nanogels Uptake

Immunofluorescence was performed as previously described [33]. 1 × 10^4^ MDA-MB-231 cells were seeded on polyethyleneimine-coated coverslips in 24-well plates and cultivated for 24 h at 37 °C. Cells were then incubated with FITC-loaded Fmoc-FF nanogels diluted in completed cell culture medium at the indicated concentrations for 1 h at 37 °C. When indicated, cellular nanogel uptake was first blocked at 4 °C for 1 h, then permitted by re-shifting the temperature to 37 °C for 10 min. At the end of each treatment, cells were fixed in 3.7% paraformaldehyde diluted in PBS pH 7.4 for 10 min at R.T. To quench paraformaldehyde, cells were incubated with a solution of 0.1 M glycine in PBS for 10 min. Cells were thus permeabilized for 10 min with PBS containing 0.1% Triton X-100. To visualize caveolae, cells were incubated with a goat anti-human HSA antibody (Invitrogen, Waltham, MA, USA, code: AB_1954616, dilution 1:200 in PBS) and a secondary anti-goat antibody coupled to Alexa Fluor™ 3 (Invitrogen, Alexa Fluor™ 350, AB_2534100, dilution 1:400 in PBS), in a humidified chamber for 1 h at RT in the dark. Coverslips were lodged on glass slides using a solution of 50% glycerol in PBS and revealed by confocal microscopy using a Leica TCS-SMD-SP5 confocal microscope (for Alexa Fluor 350: λ_exc_ = 350 nm and λ_em_ = 440 nm; for FITC λ_exc_ = 488 nm and λ_em_ = 505–600 nm), as previously described [34]. The 0.8 µm thick optical slices were acquired with a 63× or 40×/1.4 NA objective and used to generate the Z-stack galleries.

### 2.9. Statistical Analyses

All statistical analyses were conducted using GraphPad 6 software, as previously described [29]. Specific statistical tests used are reported in the figure legends. 

## 3. Results

### 3.1. Physicochemical Details of Fmoc-FF Nanogels

**Figure 1:**
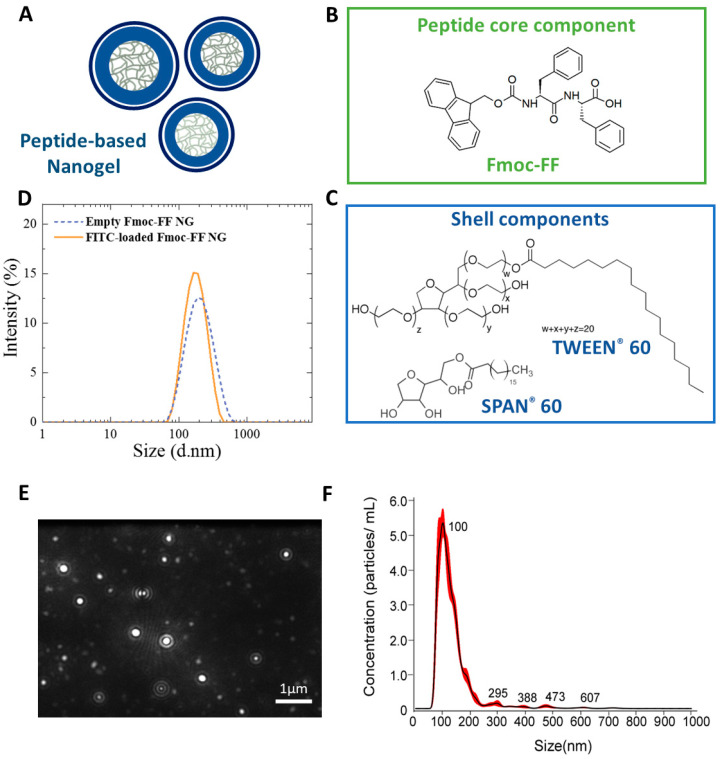
Graphical representation of nanogels and their structural characterization. (**A**) A schematic model of peptide nanogels; (**B**)chemical formulas of the core peptide; (**C**) the shell components TWEEN^®^60 and SPAN^®^60; (**D**) Size distribution of the empty and filled-FITC loaded nanogels by DLS measurements; (**E**) A representative video frame of the Fmoc-FF nanogels; (**F**) Size distribution of particles using nanoparticle tracking analysis (NTA).

Empty Fmoc-FF based nanogels were previously synthesized by us according to top-down methodology [27]. This technique consists of the submicronization of macroscopic hydrogel in the presence of the stabilizing surfactants TWEEN^®^60 and SPAN^®^60 in a 52/48 *w*/*w* ratio (total amount of 3.0·10^−5^ mol). (Figure 1A–C). FITC-filled NGs were prepared using the same procedure by simply incorporating FITC dye into the hydrogel. Structural information on both empty and FITC-filled NGs were assessed by Dynamic Light Scattering (DLS) analysis. Intensity profiles of NGs, reported in Figure 1D, reveal that both formulations have a monomodal distribution, with a mean diameter of 174 ± 82 and 184 ± 86 nm and a polydispersity index of 0.207 and 0.173, respectively. From the DLS analysis, we also measured the zeta potential (ζ) values for both formulations (−24.0 ± 0.1 and −22.0 ± 0.2 mV). The negative ζ values reflect the negative charge present on the C-terminus of the Fmoc-FF building block. Further information on the size distribution and the concentration of nanoparticles were obtained by the Nanoparticles Tracking Analysis (NTA—Figure 1E,F and Figure S1). This technique can provide a detailed characterization of the colloidal suspension by capturing the light scattered from each single particle undergoing Brownian motion. In order to identify the optimal concentration of nanoparticles in solution, the sample was 1000-fold diluted before starting with NTA analysis. This means that DLS and NTA analyses were performed under very different conditions of concentration (1 × 10^−4^ wt% and 0.1 wt% for NTA and DLS, respectively). Results of NTA measurements (Figure S1) indicate the presence of a population of nanostructures with mean diameter values (135.8 ± 5.3 nm) that are ~20% lower than those extrapolated by DLS.

This slight difference in the NG size is related to the capability of the NTA technique to accurately calculate the contribution of low and fast diffusion modes due to bigger and smaller aggregates in solution. An additional piece of information obtained by NTA analysis is the number of nanoparticles in the suspension, which in turn allows the calculation of the NG concentration (7 × 10^−10^ mol L^−1^ in the sample analyzed) and the number of peptide moieties in each nanoparticle (~2 × 10^6^).

### 3.2. Fmoc-FF Nanogels Promotes Growth Arrest of MDA-MB-231 Cells 

**Figure 2:**
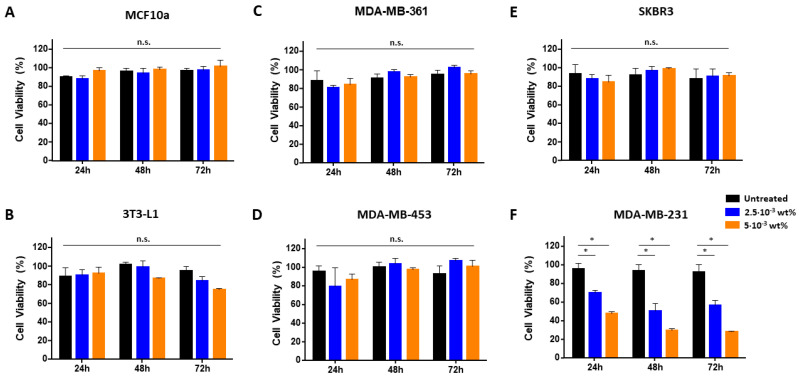
Fmoc-FF nanogels exert a cytotoxic effect on MDA-MB-231. MTT assays were conducted on MCF10a (**A**), 3T3-L1 (**B**), MDA-MB-361 (**C**), MDA-MB-453 (**D**), SKBR3 (**E**) MDA-MB-231, and (**F**) cells upon incubation for the indicated times (24, 48, and 72 h) with two different Fmoc-FF nanogel concentrations: 5 × 10^−3^ wt% (red bars) or 2.5 × 10^−3^ wt% (green bars). Cell survival is expressed as a percentage of viable cells measured in the presence of nanogels, compared to control untreated cultures. * = *p*-value < 0.05. Mann-Withey *t*-test. n.s. = not significant.

**Figure 3:**
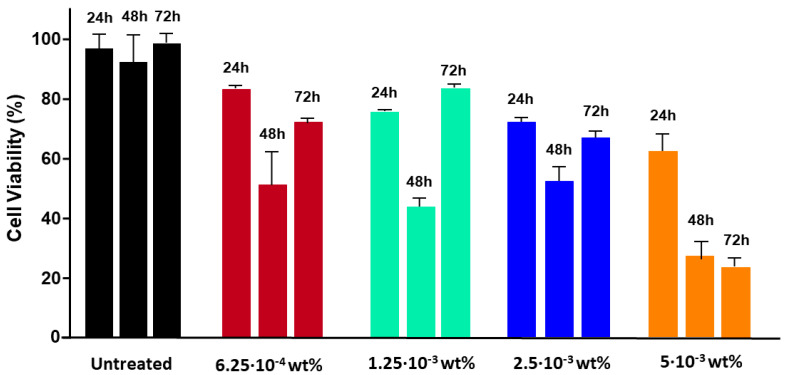
Low doses of Fmoc-FF nanogels exert a transitory cytotoxic effect on MDA-MB-231. The MTT assay was conducted on MDA-MB-231 cells treated for 24, 48, and 72 h with the indicated Fmoc-FF nanogel concentrations: 5 × 10^−3^ wt% (orange bars), 2.5 × 10^−3^ wt% (blue bars), 1.25 × 10^−3^ wt% (green bars) and 6.125 × 10^−4^ wt% (burgundy bars). Cell survival is expressed as a percentage of viable cells measured in the presence of nanogels, compared to control untreated cultures.

**Figure 4:**
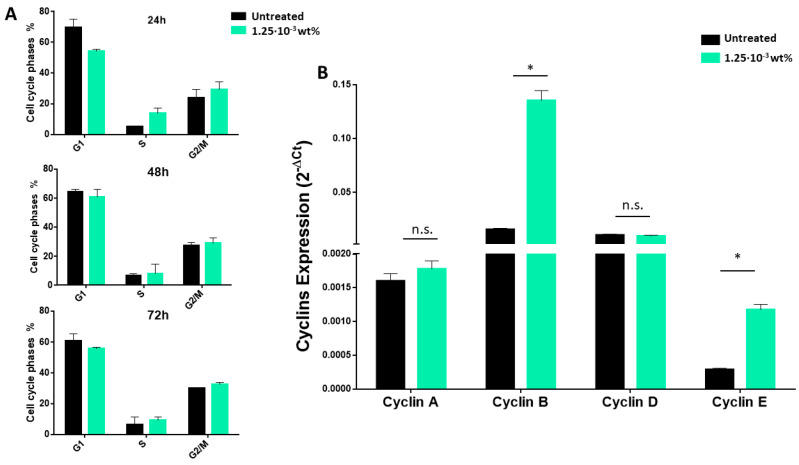
Fmoc-FF nanogels cause cell cycle arrest in MDA-MB-231 cells. (**A**) Percentage of MDA-MB-231 cells present in G0-G1, S, and G2-M phases upon treatment with Fmoc-FF nanogels at 1.25 × 10^−3^ wt% for 24 h (top panel), 48 h (middle panel), and 72 h (lower panel). The percentages are reported as the mean of three independent experiments +/− SD. (**B**). mRNA levels of the indicated cyclins in untreated (black bars) MDA-MB-231 cells and upon 24 h of treatment with 1.25·10^−3^ wt% Fmoc-FF nanogels (green bar). The relative expression was determined using the 2 − ΔCt method. Cyclins relative expression is shown as the mean +/− SD of three technical independent experiments. * = *p*-value  <  0.05, Mann Whitney *t*-test; n.s. = not significant.

The cytotoxic effect of Fmoc-FF nanogels was tested on a panel of breast cancer cells, each of which was used as a prototype of a specific breast cancer subtype: MDA-MB-361 and MDA-MB-453 (luminal breast cancer), SKBR3 (Her2 positive), and MDA-MB-231 (basal breast cancer), together with two non-tumoral cell lines, namely MCF10a (mammary gland) and 3T3-L1 (murine pre-adipocytes). As observed in Figure 2, at the end of the tested incubation periods (from 24 to 72 h) and for the tested concentrations (5 × 10^−3^ wt% and 2.5 × 10^−3^ wt%), no sign of toxicity was observed for any of the tumoral and non-tumoral cell lines (A–E), except for MDA-MB-231. For this cell line, a significant reduction in cell viability was observed after 24 h of incubation with the nanogels at a peptide concentration of either 5 × 10^−3^ wt% or 2.5 × 10^−3^ wt% and a maximum cytotoxic effect was registered after 48 h of incubation (Figure 2F and Figure S2). Interestingly, at lower peptide concentrations (1.25 × 10^−3^ wt%), nanogels caused a transitory growth arrest of MDA-MB-231, with cells recovering from cytotoxicity and regrowing after 48 h of treatment to finally acquire, upon 72 h of treatment, a viability similar to the untreated controls (Figure 3, green). This effect was also observed at an even more diluted concentration of 6.25 × 10^−4^ wt%, (Figure 3 burgundy bars). The effect of diluted solutions of nanogel suggested to them a reversible inhibitory effect on MDA-MB-231. In order to evaluate if the toxicity effect on MDA-MB-231 is due to the NG formulation or only to the externally exposed surfactants, a cell viability assay was also conducted incubating this cell line with TWEEN^®^60 alone, SPAN^®^60 alone, and a mix of them (see Figure S3). Results indicate that there is not a significant reduction in cell viability during the treatment of cells with the two surfactants or with a mix of them. The growth-arrest effect of Fmoc-FF nanogels on MDA-MB-231 was further evaluated by analyzing their cell cycle. As shown in Figure 4A (top panel), cells treated with Fmoc-FF nanogels were blocked in the S phase of the cycle. This effect was further confirmed by checking the expression levels of four regulatory cyclins. Figure 4B shows a significant increase in the expression levels of cyclins E and B in cells treated with nanogels for 24 h, consistent with a blockage of the cells in the S phase of the cell cycle.

### 3.3. Fmoc-FF Nanogel Enters MDA-MB-231 Cells via Caveolae-Mediated Endocytosis

To further investigate the cell-specific inhibitory effect of nanogels on MDA-MB-231, we aimed at the identification of the mechanism involved in Fmoc-FF nanogels intracellular uptake. Nanogels are very large structures, and, as a consequence of their dimension, their uptake should involve the endocytic machinery (endocytosis, phagocytosis, or pinocytosis). However, it was not possible to exclude a priori that the cytotoxic effect of our nanogels on MDA-MB-231 was due to the perforation (or alteration) of the Plasma Membrane (PM) promoted by the direct contact of nanogels with the lipid bilayer. 

**Figure 5:**
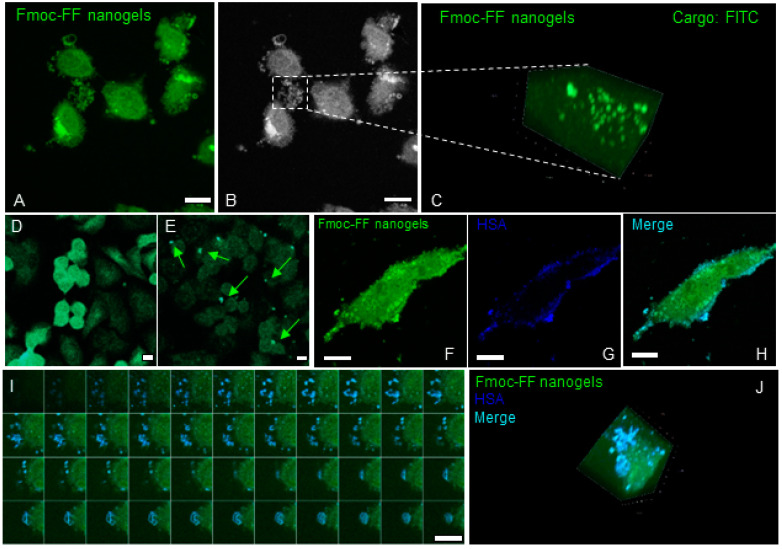
Fmoc-FF nanogels are internalized in MDA-MB-231 cells via HSA containing vesicle. (**A**) Confocal images showing the intracellular localization of FITC-loaded Fmoc-FF nanogels (green signal) upon their incubation with MDA-MB-231 cells for 1 h at 37 °C. (**B**,**C**) 3D rendering of a Z-stack gallery of confocal sections showing FITC-loaded Fmoc-FF nanogels in intracellular vesicles ~100 nm in diameter. (**D**,**E**) Confocal images showing the fluorescence of FITC-loaded Fmoc-FF nanogels incubated with MDA-MB-231 cell for 1 h at 4 °C ((**D**), note the PM staining of the FITC signal) and then switched to 37 °C for 10 min ((**E**), note the vesicular staining of the FITC signal). (**F**–**H**) Colocalization of Fmoc-FF nanogels (green, (**F**)) and HSA containing vesicles (blue, (**G**)) in MDA-MB-231 cells; the merge in panel H shows, in cyan, a region of colocalization between green and blue signals. (**I**,**J**) Gallery (**I**) and 3D rendering (**J**) of confocal z-sections showing FITC-loaded Fmoc-FF nanogels (green) colocalizing with has (blue) containing vesicles. Cyan indicates a region of colocalization between nanogels and HSA. (For all panels, magnification bar = 14 μm).

To follow nanogels entry into MDA-MB-231 cells, we loaded them with FITC, a typical fluorescent molecule used for biological assays. The MDA-MB-231 cells were incubated with FITC-loaded nanogels for 1 h at 37 °C to be then analyzed by fluorescence microscopy. As shown in Figure 5A, after 1 h of treatment, punctate FITC-fluorescent structures were visible in the cytoplasm of MDA-MB-231 cells, confirming an efficient internalization of the nanogels. The Z stacks of confocal sections revealed that Fmoc-FF nanogels containing structures present a diameter ranging from 0.1 to 1 µm (Figure 5B,C). To distinguish between nanogel permeation and endocytosis, we included a temperature block in the uptake experiment. Cells were first incubated in the presence of the nanogels at 4 °C for 1 h, then shifted to 37 °C to promote internalization (Figure 5D,E). Different from perforation and permeation, endocytic processes require the invagination and emi-fusion of the PM and these cannot occur at 4 °C. As shown in Figure 5D, the MDA-MB-231 cells incubated at 4 °C in the presence of FITC-loaded nanogels do not present internalized fluorescent structures. On the contrary, the fluorescent staining appears localized and distributed on the PM of the cells. When cells were re-shifted at 37 °C, distinct puncta appeared in the cytoplasm of the cell, confirming the involvement of an endocytic process for Fmoc-FF nanogel internalization (Figure 5E). As previously described, Fmoc-FF nanogels present a mean diameter of around 135 nm.

Their dimension should not be compatible with clathrin-dependent endocytosis and should require larger and more flexible membrane invagination like those occurring during caveolae-mediated endocytosis. Differently from phagocytosis (a process mostly occurring in specialized macrophagic cells), caveolae-dependent endocytosis is more ubiquitous and is mostly responsible for the uptake of lipid droplets and large protein complexes, like those containing albumin (HSA) [35]. In order to verify the involvement of caveolae in Fmoc-FF nanogels by MDA-MB-231 cells, we decorated them using an anti-HSA antibody. As shown in Figure 5F–H, Fmoc-FF nanogels colocalize well with HSA containing caveolae. Interestingly, in the absence of the nanogels, HSA containing caveolae appear smaller and higher in number. In the presence of the nanogels, their dimension probably increases as a consequence of the massive cargo they are internalizing. Z-stack reconstruction of confocal Z sections revealed a good colocalization between HSA and Fmoc-FF nanogels in caveolae (Figure 5I,J). 

### 3.4. Human Serum Albumin (HSA) Overload Saturates the Caveolae and Blocks the Fmoc-FF Nanogels Entering in MDA-MB-231

**Figure 6:**
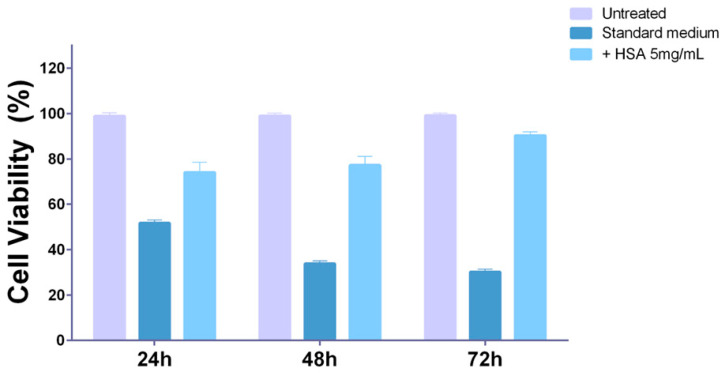
Cell viability of MDA-MB-231 cells in the presence of HSA. An MTT assay was conducted on MDA-MB-231 cells to evaluate the effects of the higher concentration of NG (5 × 10^−3^ wt%) on cell viability in standard medium (dark blue bars) and in the presence of 5 mg/mL of additional HSA (light blue bars), with respect to untreated MDA-MB-231 (light violet bars). Cell viability, expressed as a percentage of viable cells, is reported as a function of the incubation time with Fmoc-FF nanogel.

To confirm the involvement of HSA in transporting caveolae in Fmoc-FF nanogel intracellular transport, we performed a competition experiment. Briefly, we altered the composition of the cell culture medium, increasing the amount of either HSA, FBS, or glucose as a control to saturate their intracellular transport machinery. As shown in Figure S4, the addition of a double concentration of glucose (Figure S4A) or FBS (Figure S4B) did not alter the toxicity exerted by Fmoc-FF nanogels on MDA-MB-231 at a concentration of 5 × 10^−3^ wt%. Interestingly, at lower concentration of peptide nanogels (2.5 × 10^−3^ wt%), increased glucose concentration reduced their toxic effect at 24 and 48 h of incubation (Figure S4B, blue bars). Strikingly, doubling the HSA concentration in the medium reduced the toxic effects of Fmoc-FF nanogels even at the highest concentration (Figure S4C). Indeed, the viability of cells incubated with high concentrations of nanogels (50% in normal medium) increases to approximately 80% in the presence of double the amount of HSA (Figure 6 and Figure S4C). Moreover, in the presence of an excess of HSA, none of the concentrations of nanogels (1.25 × 10^−3^ wt% and 6.25 × 10^−4^ wt%) affected the viability of MDA-MB-231 (Figure S4C). These data confirm the involvement of caveolae (mostly those involved in HSA endocytosis) in nanogel uptake and suggest that their uptake can be inhibited by elevated HSA concentrations.

### 3.5. The Fmoc-FF Nanogels Selectivity toward MDA-MB-231 Results from Caveolin-1 Overexpression 

**Figure 7:**
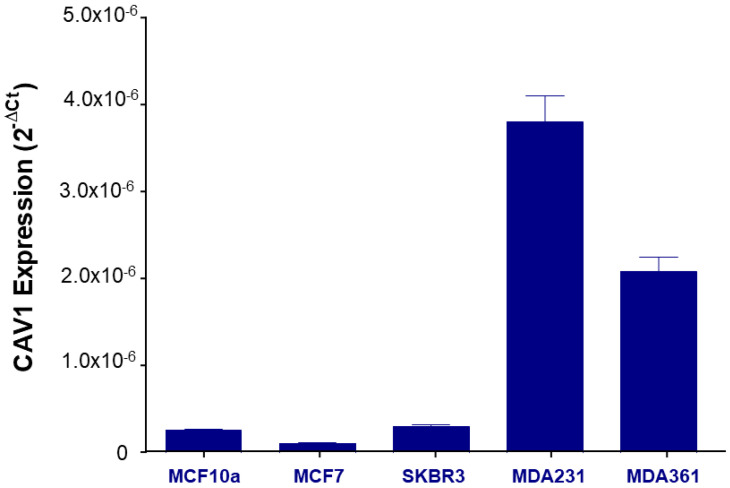
Caveolin-1 expression in breast cancer cell models. Different levels of Caveolin-1 mRNA expression in all the analyzed breast cancer cell lines. In MDA-MB-231 cells, the expression of Caveolin-1 mRNA was found to be higher (~5.0 × 10^−6^) compared to the other cell lines (values from ~0.2 × 10^−6^ to ~2.0 × 10^−6^) The relative expression was determined using the 2 − ΔCt method. Caveolin-1 relative expression is shown as the mean +/− SD of three technical independent experiments.

To further investigate the specific cytotoxicity of Fmoc-FF nanogels toward MDA-MB-231 cells, we wondered if this cell line presents a more active caveolae machinery compared to the other tested cells. Several reports have indicated that augmented caveolae-mediated endocytosis occurs in cells presenting high intracellular levels of caveolin-1 [36,37]. This protein acts as a cytosolic scaffold and helps caveolae formation by promoting membrane invagination. In order to compare the expression levels of caveolin-1 in the panel of cell lines so far used, we measured caveolin transcript by qPCR. Strikingly, the expression levels of Caveolin 1 in MDA-MB-231 were higher compared to the other cell lines, suggesting for this breast cancer clone a high rate of caveolae-mediated endocytosis (Figure 7). 

## 4. Discussion

Even though different classes of polymer-based nanogels have been reported, only rare examples of nanogels originated by using peptide building blocks have been listed in the literature until now [38,39,40]. Recently, we studied and mutually analyzed three different methodologies for the Fmoc-FF nanogel formulation, namely water/oil emulsion (W/O), top-down strategy, and nanogelling in water. Using different mixtures TWEEN^®^60 and SPAN^®^60 the effect of the Hydrophilic Lipophilic Balance (HLB) [27] value in the formulation was also evaluated, in terms of size, dimension, and stability. Our results pointed to the top-down as the best formulation strategy in terms of size (a mean diameter of 204 nm) and stability (up to one month). Additionally, an HLB of 10 was found to be the optimal value in terms of stability. The obtained nanoparticles were found to be able to encapsulate the model drug doxorubicin with a drug loading content similar to the liposomal formulations commercially available. Based on this evidence, we focused our attention on the biological fate of the nanogel formulation. Indeed, the intracellular mechanism involved in their uptake has just been postulated and is not yet fully understood. This missing information is essential to understanding the biological properties of these delivery systems and tuning their biological selectivity. The mechanisms employed by nanoparticles to enter cells are certainly affected by their structural features, which can differently interact with the biological environment and with the cell membrane. This finding is applicable also to nanogels; for instance, their dimensions, shape, and surface features (surface charge and hydrophobicity/hydrophilicity) can significatively modify the cellular uptake pathways [41]. Additional parameters to take into account to explain the uptake pathway of nanogels are the cell type and the nature of the PM such as membrane fluidity, availability of receptors. 

The cell uptake of nanogels formulated using commercial polymers has been proven to occur either by phagocytosis and/or pinocytosis, with their intracellular fate varying depending on the cell type and growing conditions [42,43]. Pinocytosis is a pathway described in almost all cell types. However, it is worth noting that cells will have a distinct profile of endocytic routes, such as macropinocytosis, clathrin-mediated endocytosis, caveolae-mediated endocytosis, or clathrin/caveolae independent endocytosis. Among them, caveolae-mediated endocytosis is the most prominent transendothelial pathway. 

Here we demonstrate that peptide based nanogels, containing the Fmoc-FF building block as an internal core and a couple of surfactants (TWEEN^®^60/SPAN^®^60), enter MDA-MB-231 cells mostly via caveolae-mediated endocytosis. This result could be expected for our NGs, taking into account some studies that predicted the mechanism of entry into the cells as a function of the nanoparticle features [35,40,44]. These studies point out that caveolae-mediated endocytosis is favored for small nanoparticles with a size lower than 200 nm, an external surface coated by polyethylene glycol (PEG), and a negative net charge. A similar mechanism of uptake has not yet been described for other nanocarriers. In 2010, Sahay et al. evaluated the cellular entry of core-cross linked polymeric micelles (cl-micelles) of poly(ethylene oxide)-b-poly(methacrylic acid) (PEO-b-PMA) copolymer in MDCK cells. In this study, the authors demonstrated that micelles enter cells selectively through caveolae-mediated endocytosis, bypass early endosomes, and reach lysosomes [45]. In 2015, Bohmer et al. studied the cellular uptake of differently modified silica-coated superparamagnetic iron oxide nanoparticles (SPIONs), which have a mean diameter of 130–150 nm [46]. In vitro studies highlighted that knockdown of Caveolin-1 reduced the endocytosis of SPIONs by HeLa cells. In the same year, Anselmo et al. described that PEG-based hydrogel nanoparticles, with a size of 200 nm, are endocytosed to a higher extent and more rapidly in immune cells (J774 macrophages), endothelial cells (bEnd.3), and cancer cells (4T1) [47].

Our results show that the intracellular machinery used by Fmoc-FF nanogels to enter the cells contributes to their selective cytotoxicity towards MDA-MB-231 cells. This cell line has been shown by us (and by others [48]) to overexpress caveolin1 and thus to possess an efficient caveolae-mediated endocytosis activity. Our results suggest that our formulation, prepared using the Fmoc-FF building block and TWEEN^®^60 and SPAN^®^60 as stabilizing surfactants, can selectively reach specific cancer cell populations, and similarly, that the delivery of cargo (like diagnostic and/or therapeutic agents) via Fmoc-FF nanogels is saturable and influenced by serum components, represented in our case by circulating serum HSA. Remarkably, to the best of our knowledge, this is the first peptide-based nanogel that demonstrates selectivity toward a cancer cell line. On the contrary, few examples of cell-selective nanogels based on polymers or proteins have been previously described. For example, in 2021, Cano-Cortes et al. reported the development of a bi-functionalized polymeric nanocarrier derivatized with a monoclonal antibody able to discriminate between cells in co-culture based on the expression levels of cell surface receptors [49]. Moreover, in 2019, Prasad Telu et al. reported the synthesis of physically cross-linked nanogels formulated using biomolecules, including DNA, protein, and biotin [50]. Due to the presence of the aptamer DNA on their surface, this nanogel demonstrated the capability of selectively recognizing the PTK7 receptor overexpressed on CCRF-CEM and HeLa cell lines. It is worth noting that although significant efforts have been put into the optimization of strategies aimed at nanogel preparation, many different aspects related to their in vivo administration (e.g., uptake mechanism, biodistribution, pharmacokinetic, and pharmacodynamics) have to be examined in depth before reaching a feasible clinical employment. Moreover, physiological aspects must be evaluated. For instance, in 2020, Solin et al. investigated the specific and nonspecific interactions between a cellulosic nanogel and human immunoglobulin G as well as bovine serum albumin (BSA) in triggering protein-surface interactions [51]. Similarly, in 2018, Wu and co-workers investigated the controlled protein adsorption and the delivery of thermosensitive poly(N-isopropylacrylamide) (PNIPAM) nanogels under different experimental conditions by using BSA as a model protein [52]. Results indicated that the protein adsorption was deeply affected by the conditions used for the study.

## 5. Conclusions

Nanogels are considered promising biomaterials for biomedicine applications since it is easy to figure them out to be used as delivery systems for drugs or diagnostic agents. However, before their feasible clinical employment, more detailed studies aimed at identifying the mechanisms of nanogel uptake, are needed at the single-cell level. By correlating the expression of caveolin with the toxicity of our nanogels in the different cell lines, we confirmed that the toxicity of nanogels toxicity increases with caveolin expression. Our data supports the hypothesis that large caveolae are necessary to engulf large nanogel particles. Cells not sensitive to nanogels are probably specialized in other forms of endocytosis, for example, chlatrin-mediated, and would be likely sensitive to smaller nanogel structures. This specific penetration mechanism confers to our NGs a selectivity toward cancer cell lines overexpressing the protein caveolin1, such as MDA-MB-231 cells. Of course, as a consequence of the heterogeneity of nanogels’ dimension, we cannot exclude that a smaller population of Fmoc-FF nanogels might enter MDA-MD-231 cells via other endocytic mechanisms, and further studies will be needed to correlate the dimension of nanogels with the endocytic machinery used to enter the cells. Analogously, to further confirm the mechanism, we also consider the possibility of performing a positive control with other commercial systems like Nab-paclitaxel that have this type of pathway on the MDA-MB-23 cells. Further study This research, focusing on the in-depth details of the cell’s uptake process exploited by the nanogel, along with its physicochemical characteristics, will open up opportunities for different biomedical applications. Furthermore, this evidence supports the ability of nanoplatforms to boost and improve the progress towards personalized medicine.

## Data Availability

Not applicable.

## Supplementary Materials

The following supporting information can be downloaded at: https://www.mdpi.com/article/10.3390/pharmaceutics15031026/s1, Figure S1: Nanoparticle tracking analysis (NTA) of peptide nanogel. (A) 2D scattergram of intensity (A.U.) vs. particle diameter (nm). (B) 2D scattergram of intensity (A.U.) vs diffusion coefficient (E4 nm^2^/s). (C) Results of the analysis (D) Representative 3D graph (particles concentration vs. intensity vs. diffusion coefficient). Figure S2. MTT assay was conducted to evaluate the effects of high concentration (5 × 10^−3^ wt%) of Fmoc-FF nanogels on the viability on the different model systems used. Cell viability was expressed as percentage of viable cells in the presence of nanogels, compared to control cells grown in their absence. Figure S3: MTT assay was conducted on MDA-MB-231 to evaluate the effects of TWEEN^®^60, SPAN^®^60 and a mix of each on cell viability. Cell survival was expressed as a percentage of viable cells in the presence of TWEEN60 and SPAN60, compared to control cells grown in their absence. Y axis reports cell survival expressed in percentage. X-axis reports the different times of incubation. Figure S4: HSA content in cell culture influences Fmoc-FF toxicity on MDA-MB-231. MTT assays were conducted on MDA-MB-231 cells treated for the reported times with the indicated dilutions of Fmoc-FF nanogels in the presence of increased concentrations of FBS (A) and glucose (B). Black bars = Untreated cells. Orange bars = Nanogels 5 × 10^−3^ wt%. Blue bars= Nanogels 2.5 × 10^−3^ wt%. (C) MTT assay was conducted on MDA-MB-231 in medium supplemented with 5 mg/mL of HSA (empty bars) respect to the standard medium (filled bars), using three different Fmoc-FF nanogel concentrations. The color bar codes are reported in the inset. Cell survival was expressed as percentage of viable cells in the presence of nanogels, compared to control cells grown in their absence.
