# Supplementary material for: Caveolin-Mediated Internalization of Fmoc-FF Nanogels in Breast Cancer Cell Lines
Source: Pharmaceutics. 2023 Mar 22;15(3):1026. doi: 10.3390/pharmaceutics15031026 (PMC10051563; doi:10.3390/pharmaceutics15031026)
Supplement: Supplementary file 1 [file pharmaceutics-15-01026-s001.zip › pharmaceutics-2274348-supplementary.pdf]

Supporting information

Caveolin-mediated internalization of Fmoc-FF nanogels in breast cancer cell lines

Giovanni Smaldone,[1,†] Elisabetta Rosa,[2,†] Enrico Gallo,[1] Carlo Diaferia,[2] Giancarlo Morelli,[2] Mariano Stornaiuolo[2,*] and Antonella Accardo[2,*]

[1]Department of Pharmacy and Research Centre on Bioactive Peptides (CIRPeB), University of Naples "Federico II", Naples, 80134, Italy.

[2]IRCCS Synlab SDN, Via Gianturco 113, Naples, 80143, Italy

[†]These Authors contributed equally to this work.

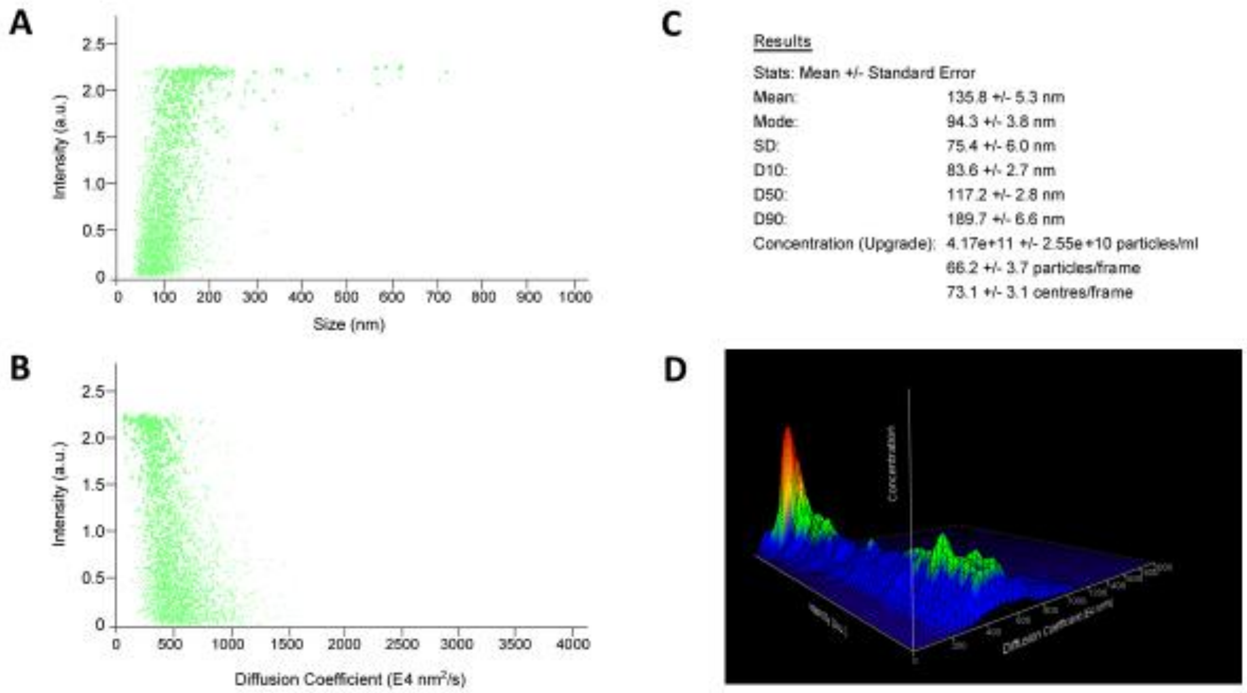

Figure S1: Nanoparticle tracking analysis (NTA) of peptide nanogel. A) 2D scattergram of intensity (A.U.) vs. particle diameter (nm). B) 2D scattergram of intensity (A.U.) vs diffusion coefficient (E4 nm^2/s). C) Results of the analysis D) Representative 3D graph (particles concentration vs. intensity vs. diffusion coefficient).

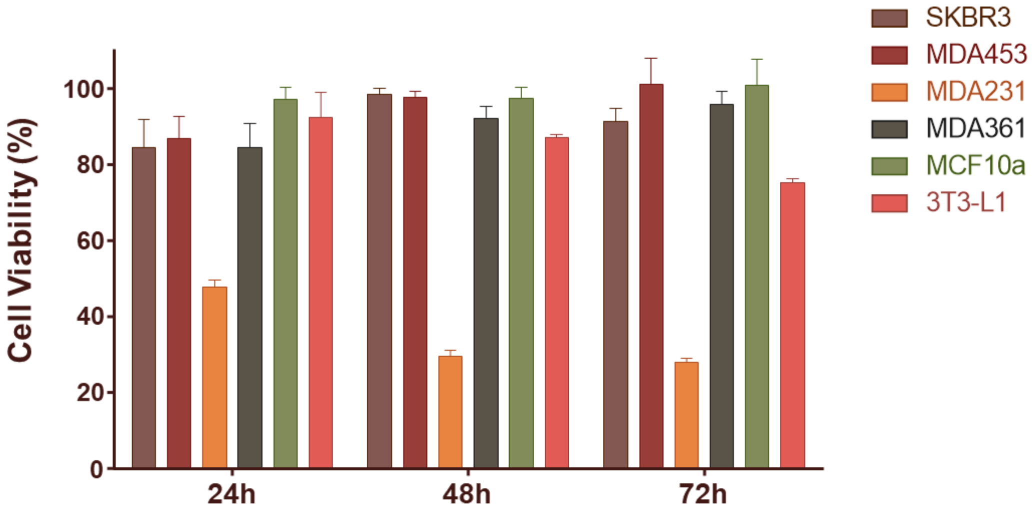

Figure S2. MTT assay was conducted to evaluate the effects of high concentration ($5 \cdot 10^{-3}$ wt%) of Fmoc-FF nanogels on the viability on the different model systems used. Cell viability was expressed as percentage of viable cells in the presence of nanogels, compared to control cells grown in their absence.

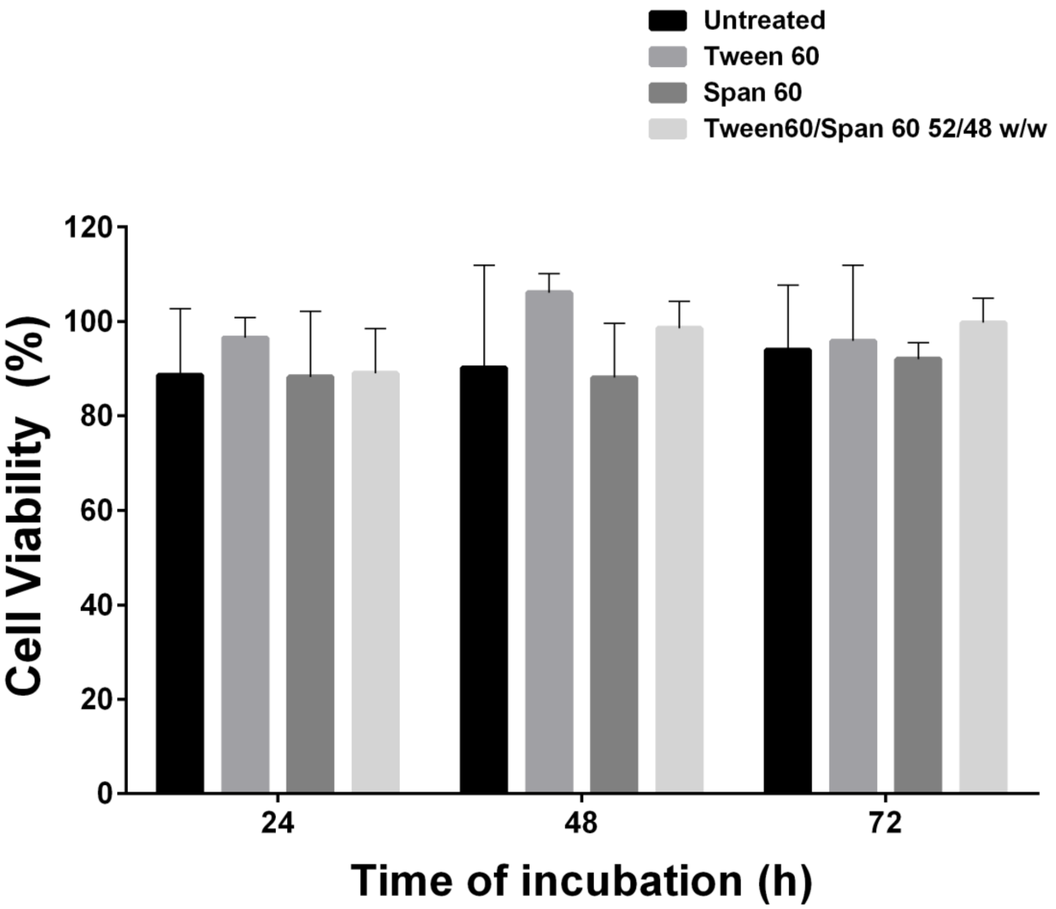

Figure S3: MTT assay was conducted on MDA-MB-231 to evaluate the effects of TWEEN®60, SPAN®60 and a mix of each on cell viability. Cell survival was expressed as a percentage of viable cells in the presence of TWEEN60 and SPAN60, compared to control cells grown in their absence. Y axis reports cell survival expressed in percentage. X-axis reports the different times of incubation.

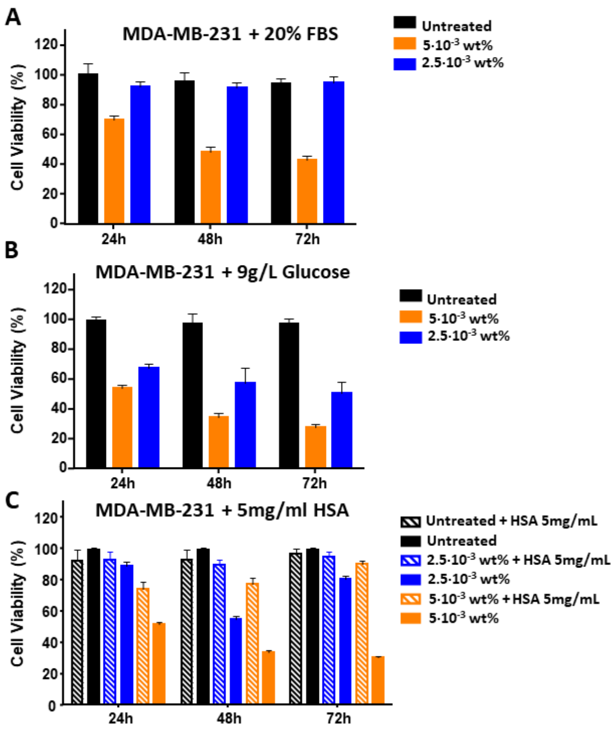

Figure S4: HSA content in cell culture influences Fmoc-FF toxicity on MDA-MB-231. MTT assays were conducted on MDA-MB-231 cells treated for the reported times with the indicated dilutions of Fmoc-FF nanogels in the presence of increased concentrations of FBS (A) and glucose (B). Black bars= Untreated cells. Orange bars= Nanogels 5·10^{-3} wt%. Blue bars= Nanogels 2.5·10^{-3} wt%. C) MTT assay was conducted on MDA-MB-231 in medium supplemented with 5mg/mL of HSA (empty bars) respect to the standard medium (filled bars), using three different Fmoc-FF nanogel concentrations. The color bar codes are reported in the inset. Cell survival was expressed as percentage of viable cells in the presence of nanogels, compared to control cells grown in their absence.
